# Identification of interaction partners of outer inflammatory protein A: Computational and experimental insights into how *Helicobacter pylori* infects host cells

**DOI:** 10.1371/journal.pone.0300557

**Published:** 2024-10-29

**Authors:** Sümeyye Akcelik-Deveci, Elif Kılıç, Nesteren Mansur-Ozen, Emel Timucin, Yaren Buyukcolak, Sinem Oktem-Okullu

**Affiliations:** 1 Department of Medical Biotechnology, Institute of Health Sciences, Acibadem University, Atasehir, Istanbul, Turkey; 2 Department of Biostatistics and Medical Informatics, School of Medicine, Acibadem University, Atasehir, Istanbul, Turkey; 3 Department of Biostatistics and Bioinformatics, Institute of Health Sciences, Acibadem University, Atasehir, Istanbul, Turkey; 4 Department of Medical Microbiology, School of Medicine, Acibadem, Atasehir, Istanbul, Turkey; University of Auckland, NEW ZEALAND

## Abstract

Outer membrane proteins (OMPs) play a key role in facilitating the survival of *Helicobacter pylori* within the gastric tissue by mediating adherence. Among these proteins, Outer inflammatory protein A (OipA) is a critical factor in *H*. *pylori* colonization of the host gastric epithelial cell surface. While the role of OipA in *H*. *pylori* attachment and its association with clinical outcomes have been established, the structural mechanisms underlying OipA’s action in adherence to gastric epithelial cells remain limited. Our study employed experimental and computational approaches to investigate the interaction partners of OipA on the gastric epithelial cell surface. Initially, we conducted a proteomic analysis using a pull-down assay with recombinant OipA and gastric epithelial cell membrane proteins to identify the OipA interactome. This analysis revealed 704 unique proteins that interacted with OipA. We subsequently analyzed 16 of these OipA partners using molecular modeling tools. Among these 16 partners, we highlight three human proteins, namely Hepatocyte growth factor (HGF), Mesenchymal epithelial transition factor receptor (Met), and Adhesion G Protein-Coupled Receptor B1 (AGRB1) that could play a role in *H*. *pylori* adherence to the gastric epithelial cell surface with OipA. Collectively, these findings reveal novel host interactions mediated by OipA, suggesting their potential as therapeutic targets for combating *H*. *pylori* infection.

## Introduction

*Helicobacter pylori* (*H*. *pylori*) colonizes the gastric epithelial cells of at least half of the world’s population and bacterial infection is associated with some gastric diseases, like chronic gastritis, and ulcer diseases. Persistent *H*. *pylori* infection with tissue damage can result in gastric cancer [1, 2].

*H*. *pylori* infection is generally acquired during childhood and in the absence of treatment with antibiotics, it persists lifelong. Although there is a high prevalence of infections worldwide, most infected individuals remain asymptomatic for a long period. 15–20% of *H*. *pylori-infected* individuals develop at least one of the associated diseases like peptic ulcer disease, gastric adenocarcinoma, and gastric mucosa-associated lymphoid tissue lymphoma at some point in their lives. The clinical outcome of *H*. *pylori* infection is believed to be influenced by multiple factors such as *H*. *pylori*-related virulence factors, host genetic predisposition, immune response, and environmental factors [3–5]. It is essential to explain how different virulence factors contribute to the pathogenesis of *H*. *pylori* and its clinical consequences to develop a vaccine and create a more potent therapeutic strategy.

The outer membrane of *H*. *pylori* consists of an asymmetric bilayer with the inner leaflet primarily composed of phospholipids and the outer leaflet containing lipopolysaccharides (LPS) and outer membrane proteins (OMPs). Inner membrane proteins are usually alpha-helical in structure, while OMPs typically have a beta-barrel shape. *H*. *pylori* also has glycosylated cholesterol embedded in its membrane [6]. OMPs have a variety of biological functions in *H*. *pylori* pathogenesis; maintaining the outer membrane structure, guaranteeing material transportation, and also playing an essential role in the process of contact with the host [7–9].

Outer inflammatory protein A (OipA), one of the most significant outer membrane proteins (OMPs) in *H*. *pylori*, plays a pivotal role in the bacterium’s virulence by facilitating its attachment to gastric mucosa cells, thereby establishing bacterial colonization during the initial stages of infection [2]. In the previous studies, it was shown that OipA-positive *H*. *pylori* strains have a stronger connection to the gastric mucosa than the strains that are OipA-negative. This highly antigenic protein increases the serum levels of interleukin-8 and the secretion of other inflammatory factors to cause neutrophil infiltration, aggravating the inflammation in the stomach. The binding of OipA to the host cell triggers an apoptotic cascade through intrinsic pathways. Pro-apoptotic pathways are activated when OipA (outer inflammatory protein A) binds to host cells and sets off a sequence of intracellular processes. This cascade likely includes the activation of additional factors involved in programmed cell death as well as signaling molecules like caspases. OipA-induced apoptosis may worsen tissue damage, impair the host’s defenses, and encourage persistent inflammation. This could facilitate bacterial colonization and lead to persistent infection by creating a suitable habitat [1, 10, 11].

Some of the *H*. *pylori* OMP interaction partners on human gastric mucosa cell layers are known and their associated host cell responses have been gradually clarified [2]. The role of OipA in the attachment of *H*. *pylori* to host cells has been confirmed. However, the interaction partners of OipA on gastric mucosa cell layers have not been defined and are the focus of this work.

In this study, we investigated the interaction partners of recombinant OipA in gastric cells by combining experimental and computational approaches. Initially, we cloned and recombinantly expressed the OipA protein from *H*. *pylori* in *E*. *coli*. Then the gastric cell membrane and surface proteins were exposed to purified recombinant OipA, and the interactions were detected using a pull-down assay.

The resulting interactors were identified by mass spectroscopic methods and further filtered by a data-driven approach. Overall, sixteen proteins and their interaction with the extracellular region of OipA were delineated by extensive computational tools including protein structure prediction, rigid- and flexible protein-protein docking, and binding free energy calculations. Combined with experimental findings, computational findings showed both novel and partly identified OipA interactions as promising drug targets for combating *H*. *pylori* infection of gastric cells.

## Materials and methods

### *H*. *pylori* G27 strain and culture conditions

The selected *H*. *pylori* G27 strain, kindly donated by Prof. Dr. Anne Mueller from the University of Zurich, Institute of Molecular Cancer Research, Switzerland, features a single plasticity area between HPG27 ORFs of 927 and 985, that comprises a large number of *H*. *pylori* specific genes compared to other strains. *H*. *pylori* is regarded as a highly fastidious organism that requires various sets of conditions for optimal growth. The bacteria were grown on plates consisting of Colombia agar (OXOID), defibrinated horse blood, and β-cyclodextrin (ChemCruz) dissolved in dimethyl sulfoxide. The plates that were supplemented with antibiotics and antifungals including 10 μg/mL Vancomycin (GeneMark), 5 μg/mL Cefsulodin (Kocak Pharma), 2.5 μg /mL polymyxin B sulfate (ChemCruz), 5 μg/mL Trimethoprim (ChemCruz), 8 μg/mL Amphotericin B (Bristol-Myers Squibb) were incubated for 3–4 days under microaerophilic conditions (gas mixture composed of 5% oxygen, 10% carbon dioxide, and 85% nitrogen). Liquid cultures were grown on Brucella broth supplemented with 10% fetal bovine serum and 10 μg/mL vancomycin for 16–24 hours. For the storage of *H*. *pylori*, − 80°C stock cultures freezing media was prepared consisting of brain heart infusion broth (BioShop^®^) containing 10% FBS (Gibco™) and 20% glycerol (SIGMA Life Science).

### DNA extraction and amplification of *oipA* gene

Bacterial genomic DNA was extracted by using Quick-DNA™ Miniprep Plus Kit (Zymo Research) according to the manufacturer’s instructions as the template. The DNA concentration was measured with a NanoDrop 2000 (Thermo Fisher Scientific, United States). The *oipA* gene was amplified by polymerase chain reaction (PCR) with the forward primer of 5’-CATTAAGCGGTGGTTTTGTG-3’ and reverse primer of 5’-AGCCAACTAAAGAGCGGTAA-3’, resulting in a product of length 1093 bp. PCR was performed in a total volume of 25 μl containing Pfu DNA polymerase (GeneMark), forward and reverse primers (0.3 μM each), 0.5 μg DNA template, dNTP (0.2 mM of each), and nuclease-free water. The PCR protocol was as follows: initial denaturation at 95°C for 5 min, 39 cycles of denaturation at 95°C for 30 s, annealing at 59.2°C for 1 min, extension at 72°C for 2 min, and a final extension at 72°C for 7 min. PCR products were analyzed under UV light with a ChemiDoc (Thermo Fisher Scientific, United States) after electrophoresis in a 1% agarose gel.

### Gene cloning and recombinant protein expression

For *oipA* gene cloning, extracted *H*. *pylori* G27 DNA samples were subjected to PCR with C-His OipA forward primer 5’-AGAAGGAGATATAACTATGATGAAAAAAGCTCTCTTACT-3’ and reverse primer 5’-GGAGATGGGAAGTCATTAATGATGGTGATGGTGGTGATGTTTGTTTTTAAAGTT-3’ that are designed to insert six histidine tag to the 3’ end of *oipA* gene. The amplified PCR product of the histidine-tagged *oipA* gene was cloned into vector pLATE11 using aLICator LIC Cloning and Expression Kit 1 (Thermo Fisher Scientific, United States) by following the kit’s instructions. The recombinant plasmid carrying the histidine-tagged *OipA* was transformed into the competent *E*. *coli* BL21 (DE3) strain with the heat shock method. After the appearance of colonies on a selective agar plate, the transformation was validated by colony PCR, and the chosen colony was used to produce C-terminal histidine-tagged OipA.

### Purification of recombinant C-His-OipA protein

A pre-culture was incubated overnight at 37°C with shaking (200 rpm). After the incubation, pre-culture was diluted 1:100 and continued to incubate until the OD600 reached 0.8. The recombinant bacteria were induced to express recombinant C-His-OipA protein by the addition of 1 mM isopropyl β-D-1-thiogalactopyranoside (IPTG, GeneMark), followed by incubation at 37°C with shaking (200 rpm), for 4 h.

After incubation, the first batch of the recombinant protein was isolated by using the B-PER™ Bacterial Protein Extraction reagent (Thermo Scientific) supplemented with 0.1 mg/mL lysozyme (GeneMark), 1U DNaseI, 0.1% Tween20, and 1X Protease Inhibitor Cocktail (Thermo Scientific) as a lysis solution. The recombinant bacterial culture pellets were centrifuged at 10.000 g for 10 minutes, treated with lysis solution for 30 minutes on ice, and followed with a sonication cycle. After the isolation, the target protein was detected in the insoluble part of the protein solution. To improve the solubility of the C-terminal His tagged OipA protein, a home-made chemical extraction solution, which consists of 6 M urea, 2 M thiourea, and 2% SDS, was used as a lysis solution for the rest of the study.

Isolated total protein from the *E*. *coli* BL21 (DE3) strain was loaded onto the SDS PAGE gel and the target protein band (between 25–35 kDa) was eluted from the gel by the passive elution method [12]. C-His-OipA protein was purified by using Dynabeads™ His-Tag Isolation and Pulldown magnetic bead system (Thermo Fisher Scientific, United States). His-tagged OipA protein isolation procedure was carried out according to kit instructions. Briefly, the protein solution was prepared with the binding and wash buffer that includes 50 mM sodium phosphate, pH 8.0, 300 mM NaCl, and 0.01% Tween™-20. Then the prepared mixture was incubated with cobalt magnetic beads at 4°C for 10 minutes. After incubation unbound proteins were washed away and bound proteins were eluted by using a His elution buffer solution containing 300 mM Imidazole, 50 mM Sodium phosphate, pH 8.0, 300 mM NaCl, and 0.01% Tween™-20.

For the refolding of purified recombinant C-His OipA protein dialysis method was used in which chemically denatured protein is refolded to sufficiently decrease the denaturant concentration and allow protein refolding. The volume of the refolding buffer that includes 0.1 mM DTT and 20 mM Tris HCl pH 8.5 was used up to 200 times the purified recombinant protein volume. Recombinant purified protein was placed into the dialysis membrane for which the length was calculated following dialysis supplier company instructions. The solution was changed 2 times with an interval of 3 hours. Twice, at three-hour intervals, the solution was changed out for a cold solution that contained just 20 mM Tris-HCl at 4°C. After that, the protein within the dialysis membrane was centrifuged for 10 minutes at 4°C at 10,000g.

### Isolation of membrane proteins

The AGS cell line derived from the human gastric adenocarcinoma (ATCC® CRL-1739™) was routinely cultivated at 37 ⁰C and 5% CO_2_ in the RPMI 1640 medium (Gibco). The growth media contained 10% fetal bovine serum (Gibco) and 1% penicillin/streptomycin (Gibco). The membrane proteins of AGS cells were isolated with the Mem-PER™ Plus Membrane Protein Extraction Kit (Thermo Fisher Scientific, United States). Briefly, 5 x 10^6^ AGS cells were lysed with the kit’s detergent, and during the centrifugation steps, the cell’s hydrophobic and hydrophilic protein phases were distributed. To maintain the 3D structure of protein mild detergents were removed by using Pierce™ Detergent Removal Spin Column, 0.5 mL kit (Thermo Fisher Scientific, United States). After taking the hydrophobic fraction of the AGS cell proteins the protein concentrations were determined by Bicinchoninic acid (BCA) assay.

### Histidine-pull-down interaction analysis

Dynabeads™ His-Tag Isolation and Pulldown magnetic bead solution (Thermo Fisher Scientific, United States) kit was used for the detection of the binding partner of OipA. The concentration ratio of OipA protein to the membrane protein mixture in the pull-down assay was established as 1:20. The refolded Histidine-tagged OipA protein was prepared with the binding and wash buffer and then incubated with cobalt-based magnetic beads as described in the purification step. After this step, unbound proteins were washed away, and then isolated AGS membrane proteins in the pull-down buffer (3.25 mM Sodium phosphate, pH 7.4, 70 mM NaCl, 0.01% Tween™-20) were mixed with bound OipA proteins on magnetic beads and incubated for 1 h at 4°C. After incubation, the protein mixture was eluted by His elution buffer. At the beginning of the experiment, a sample was prepared where all stages of the procedure were applied, except for the addition of refolded Histidine-tagged OipA protein to the magnetic beads. This sample was used as a control to identify proteins in the membrane protein mixture that might bind to the magnetic beads in the absence of OipA protein. The eluted samples were analyzed by LC-MS/MS.

### LC-MS/MS analysis

The samples, which were subjected to LC-MS/MS analysis in triplicate, included two conditions: one where the His-tag OipA protein was bound to the magnetic beads during incubation with AGS membrane proteins, and another where His-tag OipA protein was not bound to the beads (control sample). This approach allowed for a comparison of protein binding under the two conditions.

The LC-MS/MS procedure described in the ref [13] was followed. DTT and iodoacetamide were used to reduce and alkylate the proteins, respectively. Samples were diluted eight times with 50 mM ammonium bicarbonate before being incubated with trypsin overnight. Solid phase extraction (SPE) was used to desalt the digested peptides after the reaction was stopped by acidification with 10% formic acid.

Digested peptides were analyzed with 90-minute gradients in C18 nanoflow reversed-phase HPLC (Dionex Ultimate 3000,3500 RSLC nano, Thermo Scientific) combined with orbitrap mass spectrometer (Q Exactive Orbitrap HF, Thermo Scientific). Scan parameters for MS1 were; 70 000-resolution; AGC 3e6; Max IT 60 ms; Scan range 400–1500 m/z and for MS2; 17 500-resolution; AGC:5e4; Max IT:60 ms; Top 15; Isolation window:2.0 m/z; NCE: 26. Raw files were processed in Thermo Scientific Proteome Discoverer 2.3 software using Sequest search engine and human Uniprot database (Release 2016).

### Protein-protein docking

The full-length structure of OipA (accession ID: ACI27359.1) was predicted previously [14] by AlphaFold2 [15]. Both the full-length form and the mature form (excluding the signal peptide) were modeled by AF2. The mature form was used in docking analysis. The predicted structure was locally assessed based on pLDDT scores and predicted aligned error. The predicted structure of the mature form was embedded in a membrane bilayer using CHARMM-GUI and PPM2 [16–18].

The extracellular domain of the OipA (ECD) was analyzed in rigid-body docking by ClusPro [19]. Overall, a total of 20 proteins that were enlisted after MS analysis were analyzed in this docking round. Ten of the top-scoring rigid complexes were superimposed to identify the dominant binding interface of the rigid docking runs. The amino acids that contoured at least 50% of the top-scoring complexes were used as an input to the flexible docking method [20]. HADDOCK runs were performed for each complex by introducing flexibility to the interconnecting loops at the identified interface. The complexes were refined after a final iteration in water. The first representative of the top HADDOCK cluster was selected for each run. The final complexes were selected based on docking scores. ClusPro and HADDOCK are used for protein-protein docking. ClusPro primarily uses a fast Fourier transform (FFT) for global rigid-body docking [19], while HADDOCK allows for flexible docking [20]. ClusPro’s weighted score is based on electrostatics, desolvation, and van der Waals interactions. HADDOCK score is based on a weighted sum of van der Waals, electrostatics, desolvation, and restraint violations. Both scores are evaluated to find out the best binding pose by comparing their energy scores and clustering.

Three selected protein-protein complexes of OipA were predicted by AlphaFold2 multimer v3 using the Colab notebook v1.5.5 [15, 21, 22]. For all, AF2 predictions, the number of cycles was set to 12, and the top-ranking complex was relaxed in 200 iterations by the AMBER force field [23]. The interaction network in the predicted complexes was visualized by LIGPLOT Plus [24, 25]. AF2 predictions can be accessed at https://doi.org/10.5281/zenodo.13731920. For visualization of structures, Visual Molecular Dynamics (VMD) [26] and UCSF Chimera [27] have been used.

## Results

### Detection of OipA interacting partner on human gastric epithelial cell surface

**Fig 1:**
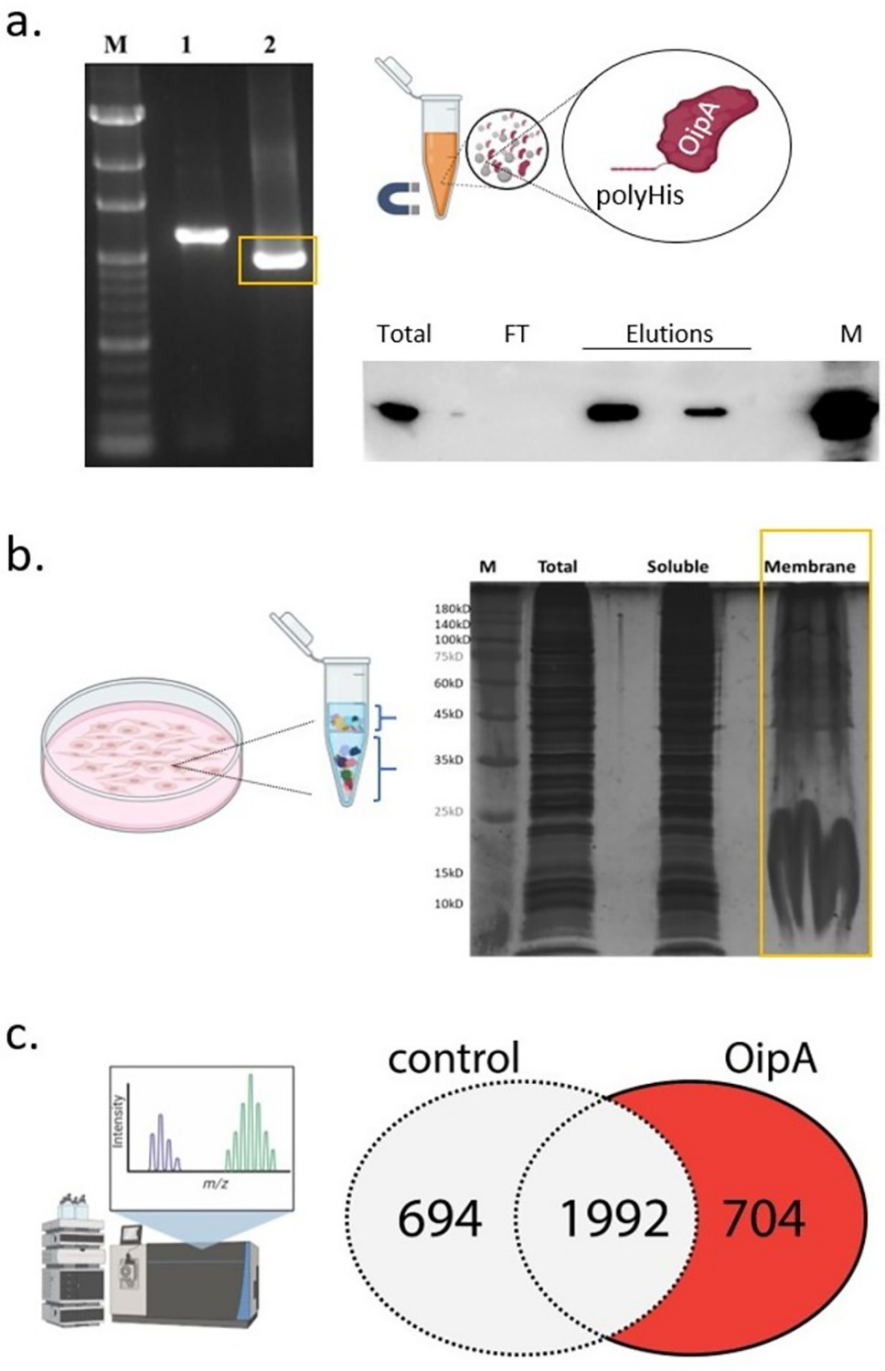
(a) Molecular cloning of the *oipA* gene (left) from *H*. *pylori* G27 genome into the aLICator LIC Cloning and Expression system. M: 100bp marker (GeneMark), 1: PCR product by priming external regions of *OipA* gene (1093 bp), 2: Full-length *oipA* gene (964 bp). After the expression of the *OipA* plasmid in *E*. *coli* BL21 (DE3), recombinant OipA was purified by cobalt magnetic beads. Western blot analysis of the purified OipA using anti-His antibody (Cell Signaling) (b) Results of membrane protein extraction from AGS cells (Mem-PER^TM^-Thermo) were shown on silver-stained SDS-PAGE gel. (c) Pull-down analysis of OipA interacting proteins from the membrane lane in (b) by LC-MS/MS. Venn diagram shows the number of identified interactors in OipA (the samples that were prepared with His-tag OipA bound beads) and control (the samples that were prepared without His-tag OipA protein) samples.

C-terminal His-tag OipA carrying plasmids were cloned using the vector: insert ratio of 1:7 and T4 DNA polymerase activity at 50°C for 30 seconds. After the transformation depending on the Sanger sequencing confirmation, the recombinant pLATE11-C-His OipA plasmid was transferred to an *E*. *coli* BL21 (DE3) competent cell. 1 mM IPTG at 37°C for 4 hours was used for the induction of protein as a result of serial optimization studies. Results of the purification of recombinant C-His-OipA protein by magnetic bead were confirmed by western blot analysis (Fig 1A). The AGS membrane proteins which keep in the interaction partner of the OipA protein were extracted by Mem-PER™ Plus Membrane Protein Extraction Kit (Fig 1B). The concentration of extracted AGS protein was measured by BCA assay and the result was 2.62 mg/mL. To identify the OipA interaction partners, the purified and refolded Histidine-tagged OipA protein was bound to cobalt-based magnetic beads (10 μg) and treated with AGS membrane proteins (200 μg). After several wash steps, the bound proteins were eluted and identified in LC-MS/MS analysis (Fig 1C).

### Identification of OipA interacting proteins

**Fig 2:**
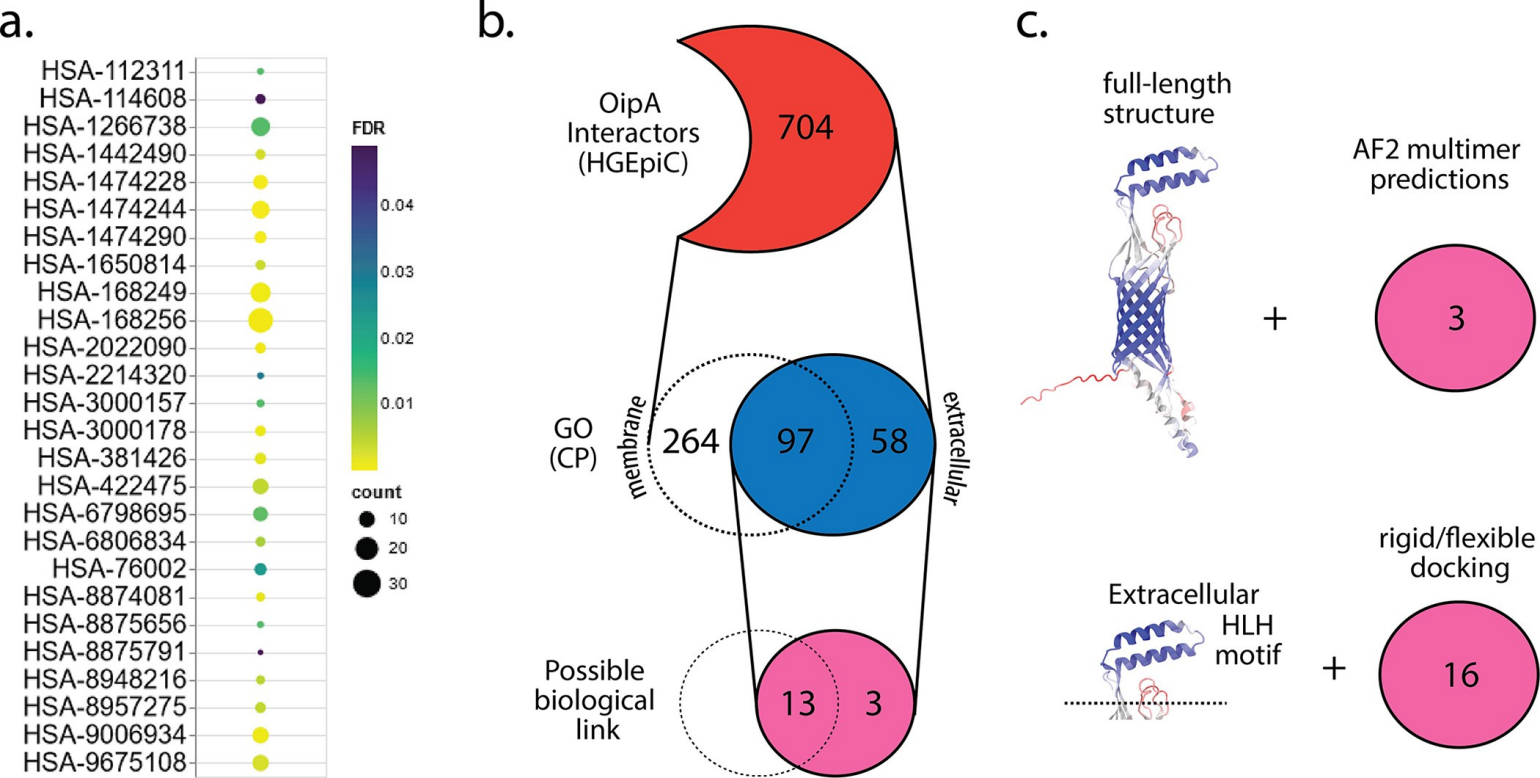
(a) shows the Reactome pathways for the proteins that are found interacting with OipA and have expected cellular compartment GO annotation. The color scale shows the corrected p-values for the analysis. S1 Table enlists the enriched pathways shown here. (b) OipA interactors were screened based on their GO cellular compartment annotation and the proteins that are either secreted or have extracellular domains/regions were considered. A biological link was established if there has been any previous study linking the identified partner and OipA and/or *H*. *pylori*. (c) shows the structural analysis conducted in this study. After the full-length structure prediction of OipA, either the full-length structure or the small extracellular portion was used in the prediction of the complex interactions that were identified in proteomics analysis.

To identify proteins interacting with OipA we analyzed the samples from pull-down assay and labeled them as OipA and control groups which indicate whether the His-tag OipA protein binds to the beads as bait at the beginning of the assay or not. Initially, we identified over 2500 distinct proteins in samples. After excluding proteins found in the control samples, we isolated 704 unique proteins as interactors of OipA. These 704 proteins were listed and subjected to subcellular localization analysis. Proteins localized to the cell membrane, either anchored to the outer membrane or spanning the bilayer, were designated as OipA interacting proteins (see S1 Table). Proteins localized to other compartments were not included in further analysis. Nevertheless, exploring the detailed atomic interactions of these proteins with OipA remains intriguing, especially since they consistently appeared as OipA interactors across all three experimental replicates analyzed by MS (Fig 2A–2C). Overall, we underline that this study represents the first effort to identify OipA interactor proteins in human gastric cells. However, it is important to acknowledge the possibility that OipA could also interact with additional partners beyond the listed targets identified in this study.

### Prediction of the full-length OipA structure

**Fig 3:**
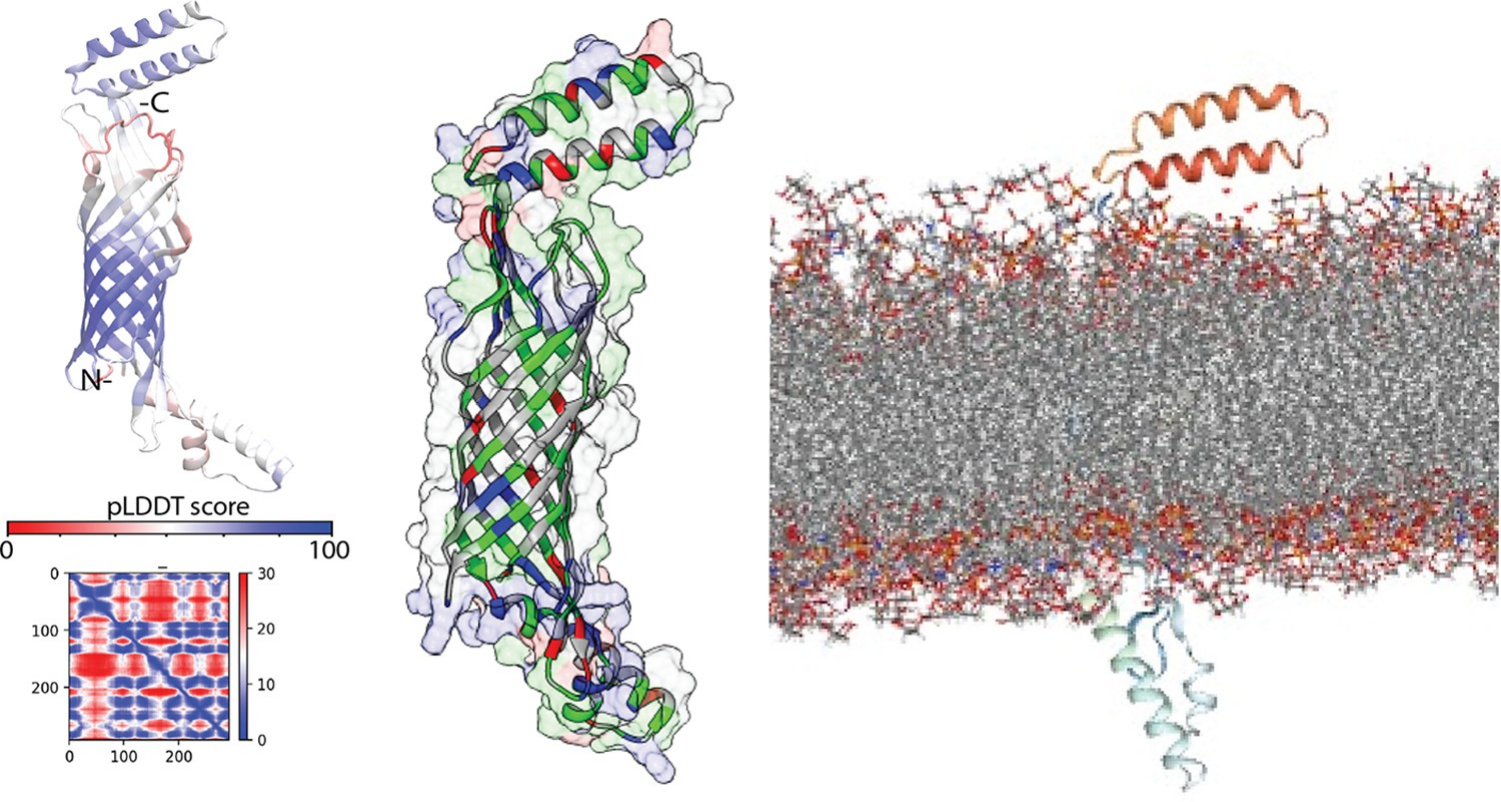
AF2 prediction of OipA structure: Left shows the full-length backbone structure colored according to the pLDDT scores, and the middle panel shows the surface contour of the same structure (white: hydrophobic, blue: basic, red: acidic, green: polar amino acids), right shows the structure embedded in a membrane bilayer formed POPE lipids. The mature OipA excluding the signal peptide was used to generate membrane-embedded form by CHARMM-GUI.

A recent study from our group that discussed the impact of the switch status of OipA on gastric diseases has reported the first prediction of the full-length OipA structure [14]. We utilized this unbound structure of OipA in the computational analysis of this study. Fig 3A shows the full-length structure with the signal peptide. We also predicted the mature form of the protein excluding the mature form that starts at the 16^th^ alanine. A comparison of the full-length and mature forms showed a Cα RMSD of 0.9 Å, implying a highly similar prediction.

The mature form of OipA has a β-barrel fold with 13 β-strands. This barrel shape when embedded in a membrane bilayer, we observed that it was completely buried in the bilayer. The barrel was lined up with highly hydrophobic amino acids, which line with the expectation of being a transmembrane protein. Despite the observation that the AF2 computed structure of OipA expectedly showed a beta-barrel structure, we noted that blind reliance on such models without any empirical data would be problematic. However, we reported that AF2 confidence metrics for this prediction of OipA structure that matches with the expected fold of the protein family were particularly acceptable. First based on the pLDDT scores, which reflect the per residue confidence of the prediction, this structure was predicted with a high accuracy i.e., a large portion of the structure has high pLDDT scores. Notably, the central beta-barrel structure has pLDDT scores higher than 70. Overall, we pursued additional docking analysis with this structure to test whether or not the listed interacting partners from the MS analysis could form a stable complex *in silico*.

Aiming to understand the role of OipA in host-cell interactions, we have focused on the potential OipA protein-protein complexes that could form in the extracellular region. Specifically, we examined the possible interactions of the helix-turn-helix motif of the OipA structure, which extends to the extracellular region (referred to as ECD), with extracellular proteins or proteins having an extracellular domain identified by MS. Through a literature search, we narrowed down the 704 proteins annotated as extracellular or having extracellular domains to 16 targets. This short list of 16 proteins was identified in the literature as potentially involved in host-interaction mechanisms of *H*. *pylori* or other microbes. For example, different microbes, such as *Listeria monocytogenes* and *Salmonella typhi*, have been shown to utilize one of these targets, Mesenchymal epithelial transition factor receptor (Met), through their outer membrane proteins to drive cell attachment or invasion [28, 29]. Specifically, the internalin protein from *L*. *monocytogenes* and the STIV protein from *S*. *typhi* were shown to interact with Met, with the former interaction also being structurally characterized (PDB ID: 2uzy) [29]. Hence, the short list of 16 targets, suggested to be involved in microbe interactions, were studied in molecular docking with OipA.

**Fig 4:**
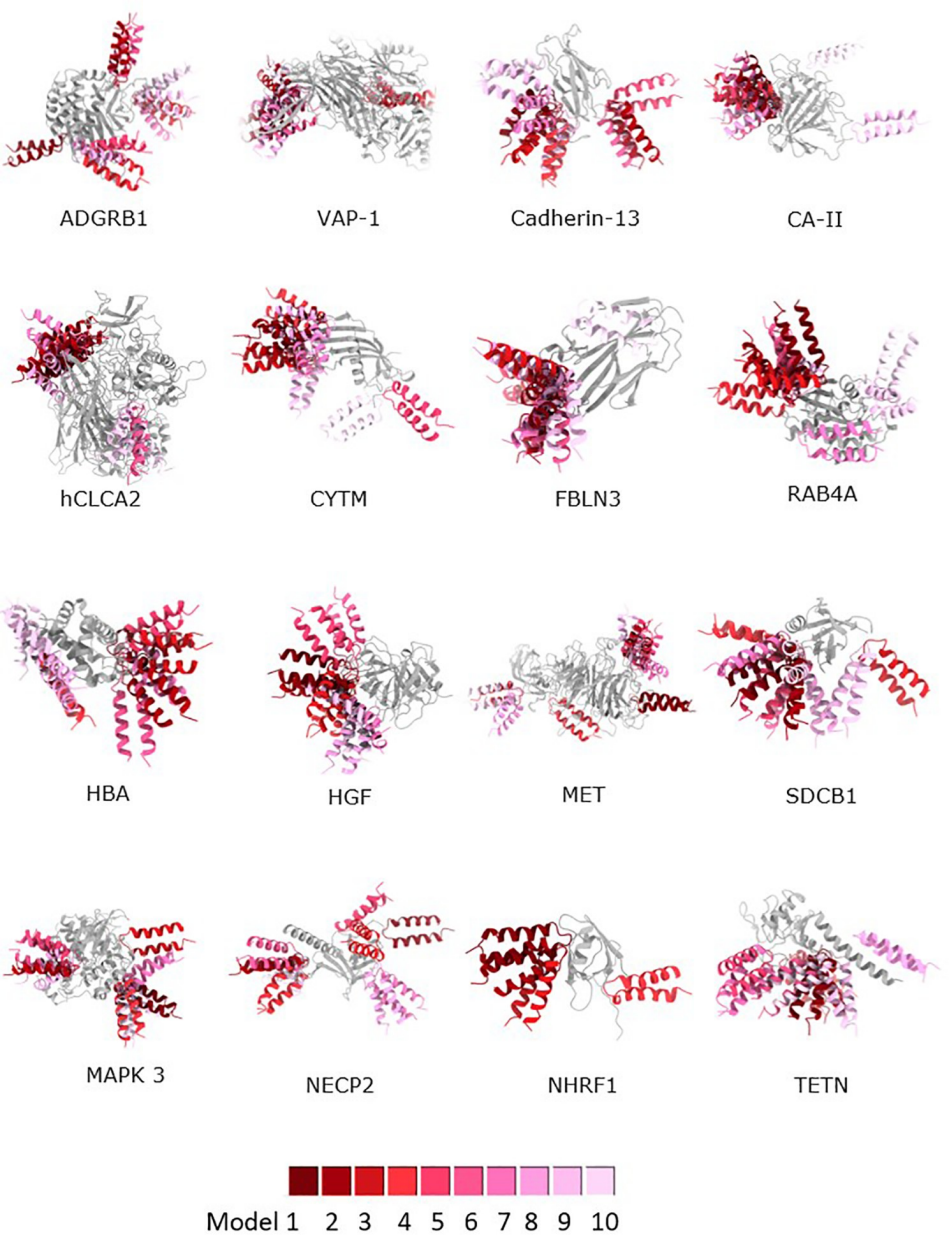
First docking analysis of the OipA interacting partners listed in Table 1 and the extracellular domain of OipA. Analysis is done by ClusPro and the top ten high-scoring models were illustrated.

The three-dimensional structures of OipA complexes with the selected 16 proteins were predicted using two protein-protein docking algorithms. The resulting OipA complexes were visualized in Fig 4. Rigid-body predictions of these complexes indicated that the bound complexes consistently shared the same interface, suggesting a convergence of rigid-body results. Subsequently, flexible docking runs were conducted to refine the docked complexes, as shown in Fig 4. Docking scores obtained from this second round of flexible docking are listed in Table 1.

**Table 1:** List of possible OipA interactors that were analyzed in molecular docking.

UniProt	Gene name	Structure (source)	Score*	Reference
P00918	CA-II	1BCD (PDB)	-133.4±1.5	[30, 31]
O14514	AGRB1	ADGRB1 (AF2)	-82.7±1.7	[32]
Q16853	VAP-1	4BTX (PDB)	-131.9±0.3	[33]
P05452	TETN	1HTN (PDB)	-69.1±1.3	[34]
Q15828	CYTM	4N6L (PDB)	-65.8±2.7	[35]
Q9UQC9	CLCA2	Q9UQC9 (AF2)	-119.0±6.8	[36, 37]
P69905	HBA1	2DN1 (PDB)	-121.4±2.4	[38]
P20338	RAB4A	2BMD (PDB)	-93.6±2.9	[39]
O14745	NHRF1	4Q3H (PDB)	-90.4±1.7	[40]
Q12805	FBLN3	Q12805 (AF)	-93.1±0.6	[41]
P08581	Met	2UZX (PDB)	-132.1±2.3	[29, 42]
Q9NVZ3	NECP2	Q9NVZ3 (AF)	-90.8±2.2	[43]
P14210	HGF	4O3T (PDB)	-103.4±0.8	[44–46]
O00560	SDCB1	1NTE (PDB)	-68.6±0.4	[47]
P55290	CAD13	2V37 (PDB)	-64.9±4.0	[48]
P27361	MAPK 3	4QTB (PDB)	-118.6±5.1	[49, 50]

*HADDOCK score is in kcal/mol.

**Fig 5:**
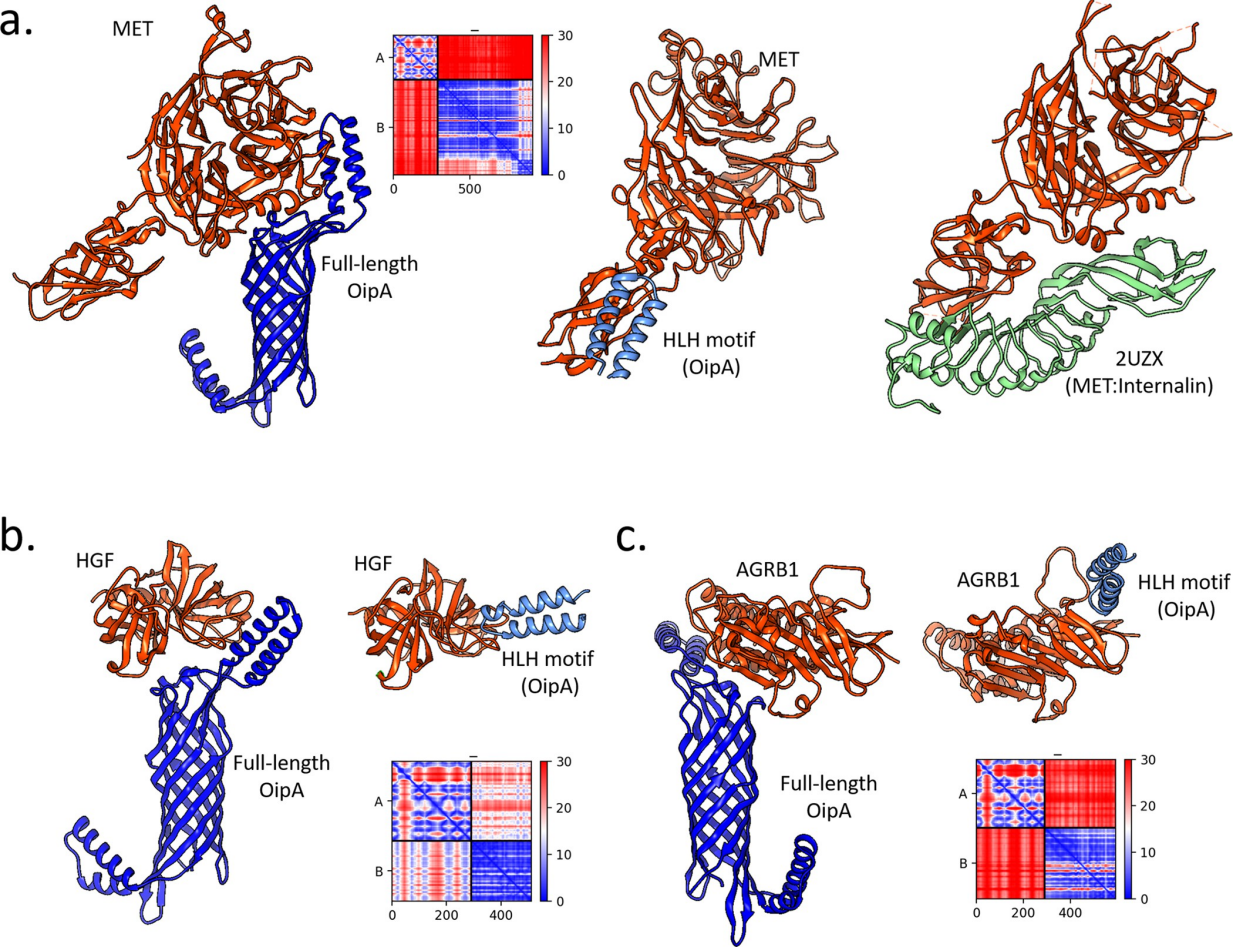
Results of OipA complexes that were predicted either using the full-length structure (AF2) or the helix-loop-helix (HLH) motif (docking). (a) Panel shows the predicted complexes with the Met. The crystal structure that captured Met and *L*. *monocytogenes* internalin protein. (b) Panel shows the complexes formed with HGF and (c) shows formed with AGRB1.

We also analyzed the interaction between the full-length OipA and its three partners: Hepatocyte growth factor (HGF), Mesenchymal epithelial transition factor receptor (Met), and Adhesion G Protein-Coupled Receptor B1 (AGRB1). To model the full-length complex structure, we utilized AF2 multimer [22, 51]. The resulting complexes differed somewhat from the predictions made for the extracellular helix-loop-helix (HLH) motif of OipA (Fig 5A—Met, 5B—HGF, 5C—AGRB1). Notably, the binding of full-length OipA to Met occurred at the extracellular domain of Met (Fig 5A). In contrast, both predictions for the HGF complex overlapped, highlighting a small cleft in the HGF structure (Fig 5B). However, the predictions did not show the same OipA surface for the full-length prediction; the binding interface consisted of the small extracellular HLH motif along with the outer mouth of the β-barrel structure (Fig 5B). Overall, the full-length predictions, compared with the docking of the small helix-turn-helix motif of OipA, provided insights into the possible complexes of OipA with several key proteins, offering a plausible structural explanation for OipA’s role in adhesion.

A detailed view of the protein interactions occurring at the interface of predicted complexes was given in the supplementary information (S1-S4 Fig in S1 File). Essentially, we compared the PDB complex formed by Met kinase and internalin, an invasion protein from another bacterium *L*. *monocytogenes* (PDB ID: 2uzx) with the AF2 predicted complex of Met-OipA. Although internalin which adopts extensive beta-sheet conformation, binds to a larger surface on the Met compared with that of OipA, we spotted a partial overlap between the binding surface of these two bacterial proteins in their Met complexes. Specifically, two residues on Met, Y328, and F332 are adjacent helix residues shown in S5 Fig in S1 File and were found to interact with internalin (S2 Fig in S1 File) and with OipA (S3 Fig in S1 File), respectively.

## Discussion

*H*. *pylori* forms a strong interaction with gastric epithelial cells [52] and has developed mechanisms to survive and cause infection in the harsh acidic environment of the stomach. The pathogenesis of *H*. *pylori* infection and disease outcomes are influenced by host genetics, environmental factors, and bacterial virulence factors [53]. These virulence factors play a crucial role in adherence, colonization, and activation of the host immune response. Specifically, the adherence of *H*. *pylori* to the mucus layer of the gastric epithelium is essential for initiating colonization, which leads to infection [8].

The large outer membrane protein (OMP) family of the bacterium includes proteins that are specifically involved in attachment [54]. These outer membrane proteins interact with cellular receptors, which help the bacteria survive in acidic environments, resist mucus, and withstand stomach exfoliation [54, 55]. OipA, one of the outer membrane proteins, plays a crucial role in bacterial pathogenesis by attaching to gastric epithelial cells. The functional status of OipA is determined by a slipped strand mispairing mechanism, and patients infected with *H*. *pylori* with a functional OipA have carry a higher risk of developing gastric cancer [56]. OipA facilitates a strong attachment between the bacteria and gastric epithelial cells, either directly or through interaction with surface proteins during colonization [52]. While OipA has been recognized as a vital virulence factor of *H*. *pylori*, its interaction partners in gastric epithelial cells have not been elucidated yet. In this background, we conducted this study to find the interaction partners of OipA in human gastric epithelial cell lines using a pull-down assay followed by LC-MS/MS analysis.

Proteomic analysis revealed 704 unique binding partners of OipA in gastric epithelial cells. From this extensive list, we selected a small set of OipA binders based on subcellular localization and biological relevance. This set includes Hepatocyte Growth Factor (HGF), Hepatocyte Growth Factor Receptor (Met), Carbonic Anhydrase II (CA-II), Adhesion G Protein-Coupled Receptor B1 (AGRB1), Vascular Adhesion Protein 1 (VAP-1), Tetranectin (TETN), Cystatin-M (CYTM), Calcium-Activated Chloride Channel Regulator 2 (CLCA2), Hemoglobin Subunit Alpha (HBA1), Ras-Related Protein Rab-4A (RAB4A), Na(+)/H(+) Exchange Regulatory Cofactor (NHRF1), EGF-Containing Fibulin-Like Extracellular Matrix Protein 1 (FBLN3), Adaptin Ear-Binding Coat-Associated Protein 2 (NECAP2), Syntenin-1 (SDCB1), Cadherin-13 (CAD13), and Mitogen-Activated Protein Kinase 3 (MAPK3). Each of these 16 protein partners was extensively analyzed using molecular modeling methods, resulting in 16 distinct complex structures of OipA.

The quality of the computed structures that were not derived from any experimental data should be properly evaluated. Among the recent artificial intelligence methods that were developed to predict protein structure, most of the tools provide a means of quality evaluation. The method that was employed here, AF2, essentially uses a metric for assessment of the reliability of the predicted Cα positions [57], which is called the predicted local difference distance test (pLDDT) score. Given the availability of such a measure at the residue level, one could assess the quality of the predicted OipA backbone structure. From this regard, we considered the pLDDT scores of each amino acid for the OipA structure and found out that the full-length AF2 structure of OipA having most of the amino acids with the pLDDT scores higher than 70 (Fig 3). Especially, the pLDDT-based scores were much higher at the core β-barrel region while predictably the confidence scores of the loop regions were lower. Nonetheless, we underscore that the first structural modeling of the OipA structure would open new doors to understanding the molecular mechanism of the protein in the pathogenesis of *H*. *pylori*.

OipA or any of its other close relative has not been structurally characterized because they localize in the outer membrane of *H*. *pylori*, rendering their experimental characterization challenging. Due to the absence of another structure with an apparent homology to OipA, comparative modeling techniques may fail or produce unreliable results. As such, during this study, we have implemented both homology modeling and threading approaches to predict the 3D structure of *H*. *pylori*’s OipA using SWISS-MODEL [58] and ITASSER [59], respectively. However, comparative modeling results were not satisfactory to show a barrel-like structure in line with the characteristic fold of this family of proteins [60]. In line with the current advances in deep-learning-based protein structure prediction methods, we utilized AlphaFold2 (AF2) to predict the OipA structure [61]. The predicted structure has most of the amino acids with pLDDT scores higher than 70 (Fig 3). Notably, the core β-barrel region, exhibiting the highest pLDDT scores approaching 100, was accurately predicted, while the loop regions were predictably assigned lower confidence scores below 50. Despite these limitations, we emphasize that this initial structural modeling of OipA provides valuable insights into the structural mechanisms underlying the involvement of OipA in *H*. *pylori* pathogenesis [14].

Structure-based modeling also provided novel insights about the OipA complexes with hepatocyte growth factor (HGF) as well as its receptor (Met). Essentially, these two proteins are tightly linked with infectious diseases as several pathogens have been found to hijack the HGF-Met system, affecting the expression of HGF or Met through the use of their pathogenic factors [62]. As a result, the HGF-Met system stands out as both a therapeutic target and a marker for infections [62]. The HGF/c-Met signaling pathway is also associated with the polarity of epithelial cells, which affects their susceptibility to pathogenic microorganisms [63]. Notably, one of the significant virulence factors, CagA, targets the HGF/c-Met signaling pathway, enhancing cell scattering—a mitogenic response indicator in *H*. *pylori*-infected gastric epithelial cells [64–66]. Additionally, another Gram-negative human pathogen, *L monocytogenes*, utilizes the HGF-Met axis to invade host cells. Specifically, a structural study revealed a crystal complex structure between internalin, an InlB protein of the pathogen, and Met [67]. Comparing this structure with our OipA-Met complexes showed a partial overlap in the binding interfaces of both models (Fig 5A). Altogether, the pivotal role of the HGF-Met signaling pathway in the progression of infectious diseases, combined with our proteomic analysis, suggested a binding activity of recombinant OipA towards Met on the gastric epithelial cell surface. Our structural predictions further imply that this interaction occurs at a similar Met surface utilized by *L*. *monocytogenes*. Thus, in addition to the established literature on HGF/Met and CagA interactions, our findings provide novel insights into how *H*. *pylori* infects its host through the HGF/Met-OipA interaction, further reinforcing the potential of HGF/Met as a therapeutic target for this infection.

Another binding partner of OipA, Adhesion G protein-coupled receptor B1 (AGRB1), which is a phosphatidylserine receptor, was identified as mediating the binding and engulfment of Gram-negative pathogens [32, 68]. In this study, we also explored how this protein interacts with OipA using both full-length and partial OipA structures. Our analyses suggested that the AGRB1 interaction likely occurs on the outer part of OipA. The full-length model showed AGRB1 attaching to the extracellular part of OipA, particularly exhibiting a flexed HLH motif (Fig 5C).

While the experimental section of this study including heterologous protein expression, in-vitro pull-down assays, and LC-MS approaches provides valuable insights into the interacting partners of OipA, they may not conclusively verify direct protein interactions. Validating the short-listed *in-silico* predictions would be required to ultimately reveal the molecular machinery behind the OipA role in *H*. *pylori* infection.

The binding between the OipA protein and its candidate interaction partner is a crucial step in the attachment of *H*. *pylori* to the gastric mucus layer. Targeting these interaction partners could be an effective strategy to prevent infection. Therefore, we conclude that the identified interaction partners of OipA, determined through proteomic analysis, hold significant promise for future studies aimed at understanding the molecular mechanisms of OipA in *H*. *pylori* infection.

## Supporting information

S1 Table List of OipA interacting proteins. (XLSX)

S1 Raw images Raw images for agarose gel and western blot. (PDF)

S1 File Protein-protein interactions within the full-length OipA. (PDF)
