# Supplementary material for: Identification of interaction partners of outer inflammatory protein A: Computational and experimental insights into how Helicobacter pylori infects host cells
Source: PLoS One. 2024 Oct 29;19(10):e0300557. doi: 10.1371/journal.pone.0300557 (PMC11521304; doi:10.1371/journal.pone.0300557)
Supplement: S1 Raw images — (PDF) [file pone.0300557.s002.pdf]

**Original image for agarose gel shown in Fig. 1a.**

M External *OipA* Full-length *oipA*

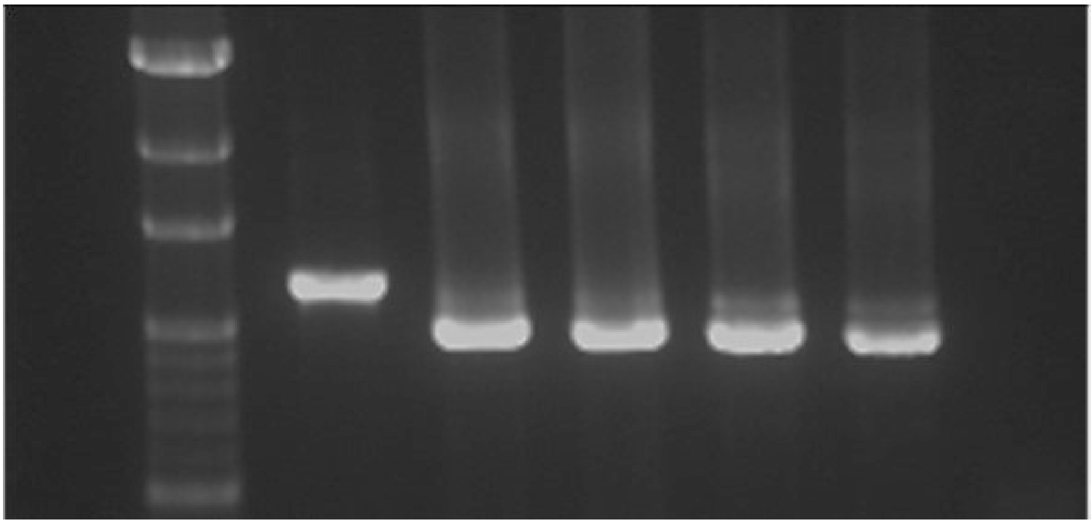

Lanes: 1: 100bp marker (GeneMark); 2: PCR product by priming external regions of *OipA* gene (1093 bp); 3-6: Full-length *oipA* gene (964 bp).

**Original image for Western blot shown in Fig. 1a.**

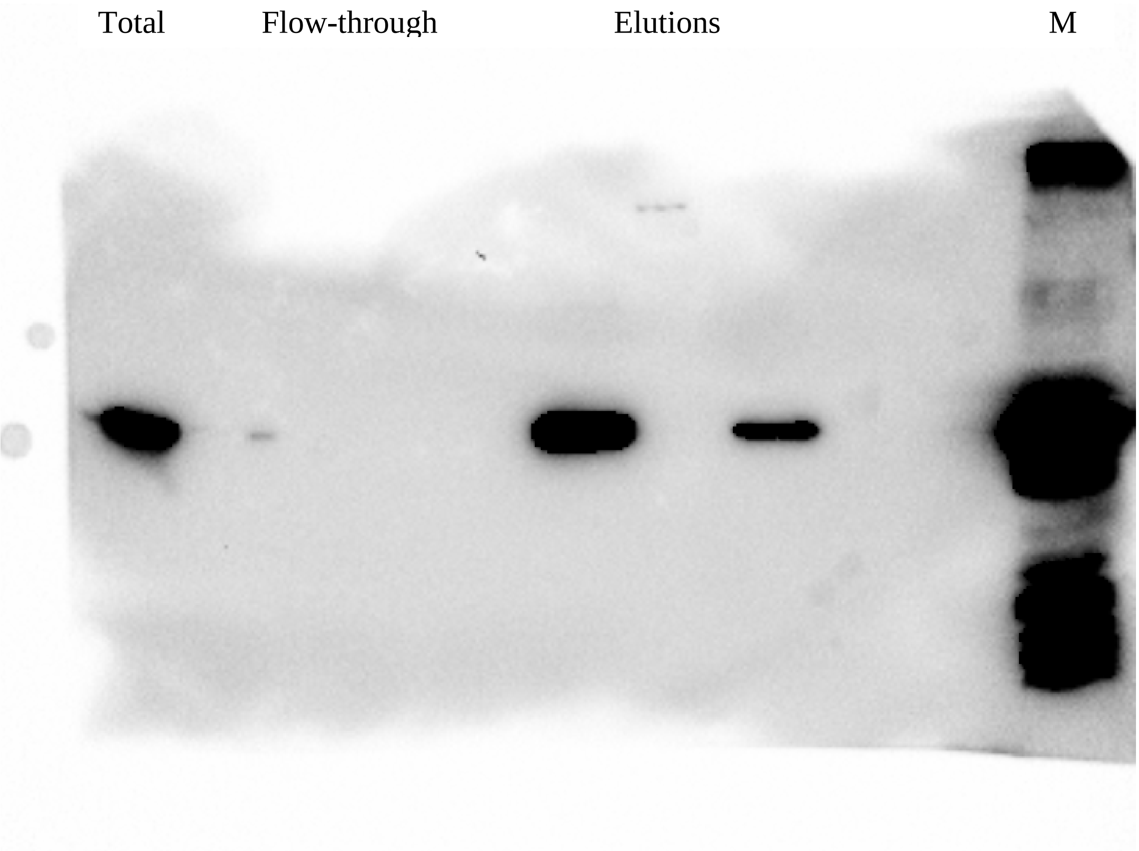


Lanes: 1: Total protein; 2-4: Flow-through; 5,7: Elutions; 10: Marker

**Original image for SDS-PAGE gel shown in Fig. 1b.**

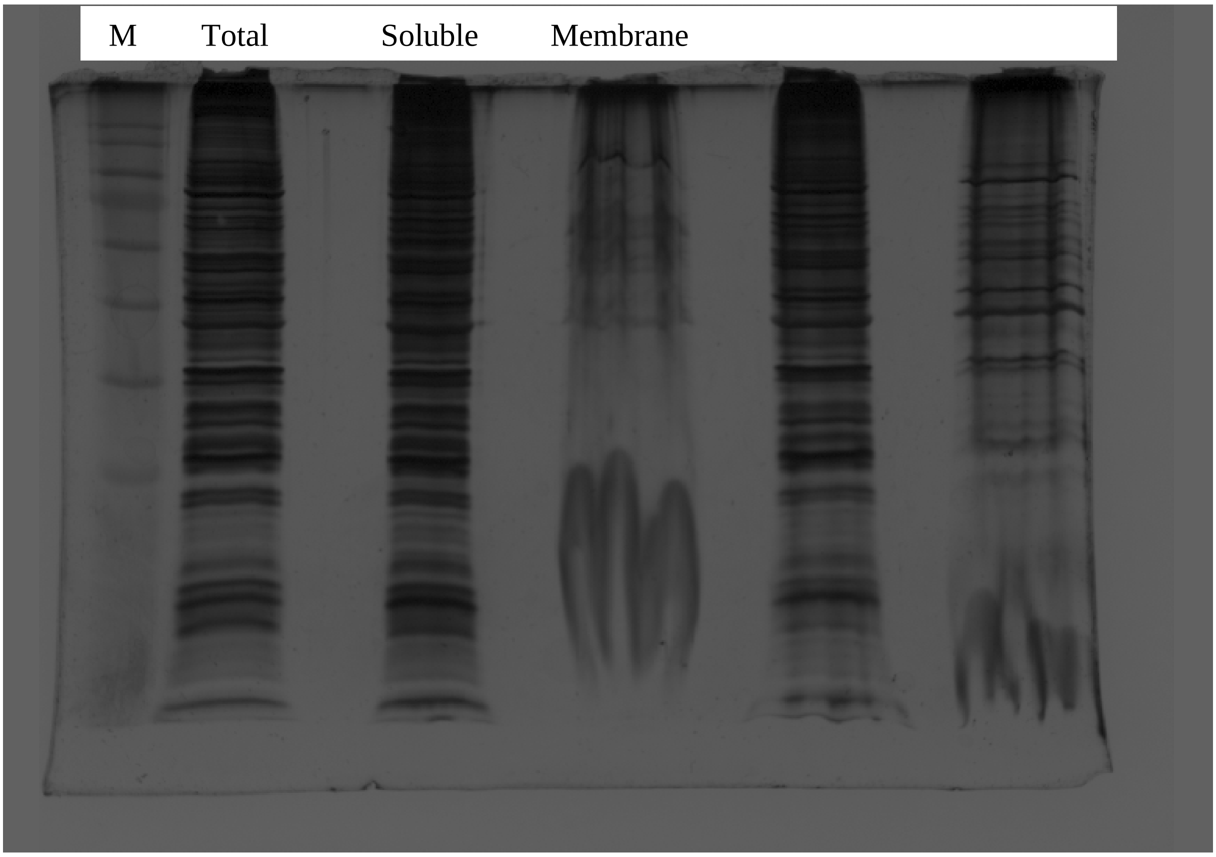


Lanes: 1: Marker; 2: Total protein; 4: Soluble protein; 6: Membrane protein
