# Supplementary material for: Identification of interaction partners of outer inflammatory protein A: Computational and experimental insights into how Helicobacter pylori infects host cells
Source: PLoS One. 2024 Oct 29;19(10):e0300557. doi: 10.1371/journal.pone.0300557 (PMC11521304; doi:10.1371/journal.pone.0300557)
Supplement: S1 File — (PDF) [file pone.0300557.s003.pdf]

Supplementary Information

Identification of Interaction Partners of Outer Inflammatory Protein A: Computational and Experimental Insights into How *Helicobacter pylori* Infects Host Cells

Sümeyye Akcelik-Deveci[1], Elif Kılıç[1], Nesteren Mansur-Ozen[1], Emel Timucin[2,3], Yaren Buyukcolak[1], Sinem Oktem-Okullu[4*]

Affiliations

[1]Department of Medical Biotechnology, Institute of Health Sciences, Acibadem University, Atasehir Istanbul, Turkey

[2]Department of Biostatistics and Medical Informatics, School of Medicine, Acibadem University, Atasehir, Istanbul, 34684, Turkey

[3]Department of Biostatistics and Bioinformatics, Institute of Health Sciences, Acibadem University, Atasehir Istanbul, 34684, Turkey

[4]Department of Medical Microbiology, School of Medicine, Acibadem, Atasehir, Istanbul, 34684, Turkey

[*]Correspondence: sinem.oktem@acibadem.edu.tr; sinemoktemokullu@gmail.com

Telephone: +90 554 587 4828

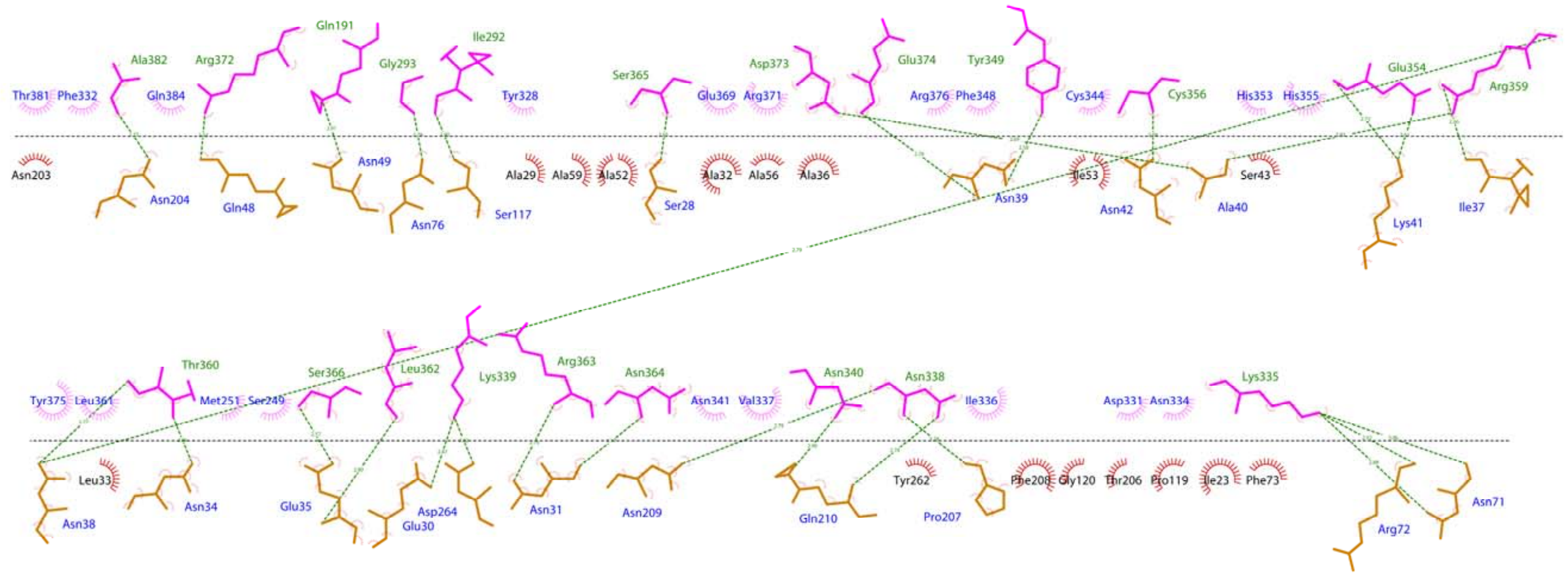

Fig. S1. Protein-protein interactions within the full-length OipA and Met complex predicted by AF2. Residues from OipA are shown at the bottom (brown) and those from Met at the top (pink). Hydrogen bonds were shown as green lines. Hydrophobic interactions were shown circles.

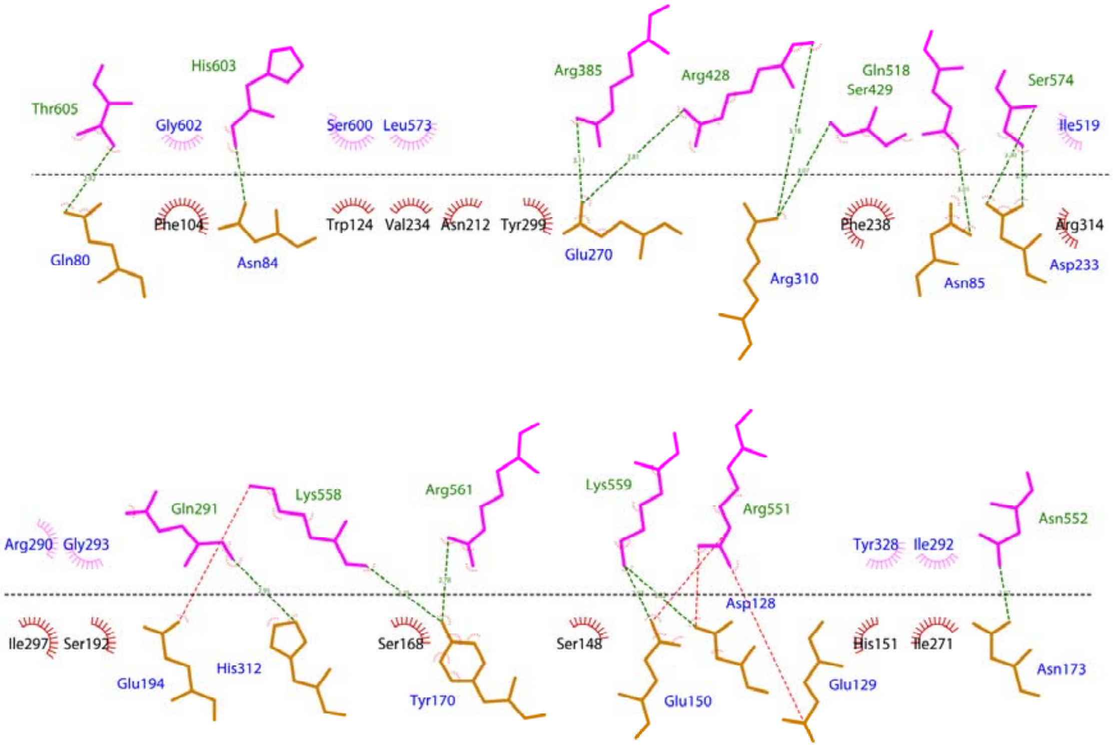

Fig. S2. Protein-protein interactions within the PDB complex (2uxz) formed by Met and the invasion protein internalin B of *Listeria monocytogenes*. Residues from internalin are shown at the bottom (brown) and those from Met at the top (pink). Hydrogen bonds were shown as green lines. Hydrophobic interactions were shown circles.

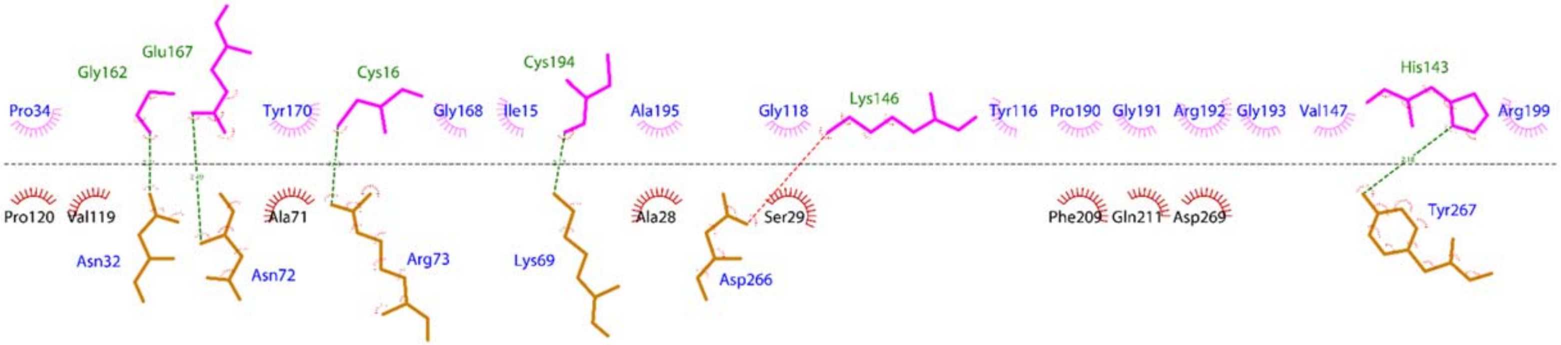

Fig. S3. Protein-protein interactions within the full-length OipA and HGF complex predicted by AF2. Residues from OipA are shown at the bottom (brown) and those from HGF at the top (pink). Hydrogen bonds were shown as green lines. Hydrophobic interactions were shown circles.

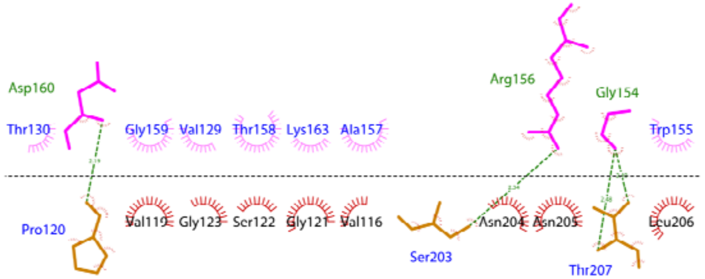

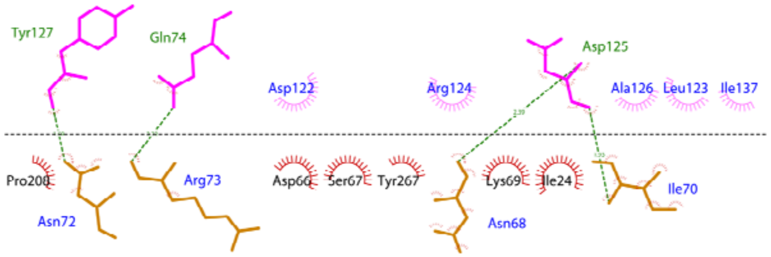

Fig. S4. Protein-protein interactions within the full-length OipA and AGRB1 complex predicted by AF2. Residues from OipA are shown at the bottom (brown) and those from AGRB1 at the top (pink). Hydrogen bonds were shown as green lines. Hydrophobic interactions were shown circles.

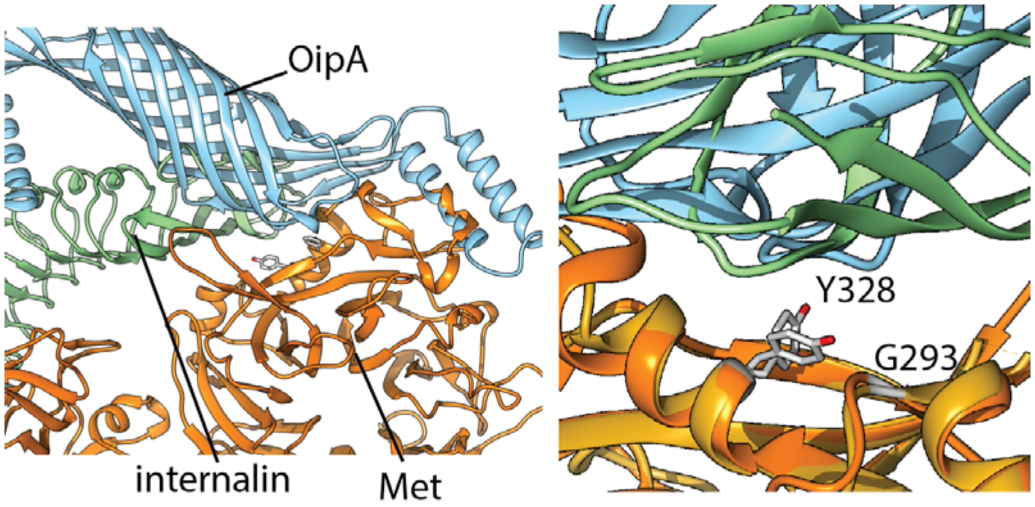

Fig. S5. Superimposed complexes of Met-OipA and Met-Internalin (PDB ID: 2uzx) showing a partial overlap in their binding interface. Particularly two residues G293 and Y328 were found at the binding interface of both complexes (Fig. S1-2).
